# Selective through-holing of anodic porous alumina membranes with large area

**DOI:** 10.1039/c8ra07646d

**Published:** 2018-11-14

**Authors:** Takashi Yanagishita, Yuki Okubo, Toshiaki Kondo, Hideki Masuda

**Affiliations:** Department of Applied Chemistry, Tokyo Metropolitan University 1-1 Minamiosawa Hachioji Tokyo 192-0397 Japan yanagish@tmu.ac.jp

## Abstract

Anodic porous alumina membranes with site controlled through-holes were prepared by the formation of a masking layer on the surface of anodic porous alumina and subsequent selective second anodization in concentrated sulfuric acid to form a readily soluble layer. After the anodization, the residual Al substrate was removed, and the highly soluble alumina layer formed in concentrated sulfuric acid was dissolved selectively by wet etching. An advantageous point of this process is the controllability of the pattern of through-holes, and the preparation of large samples with selective through-holes is possible. The pattern of through-holes was controlled by changing the mask pattern formed on the surface of anodic porous alumina. The alumina membranes obtained by this process are expected to be used for various applications that require porous alumina membranes with site controlled through-holes.

## Abstract 2

Anodic porous alumina membranes with selective through-holing.

## Graphical abstract

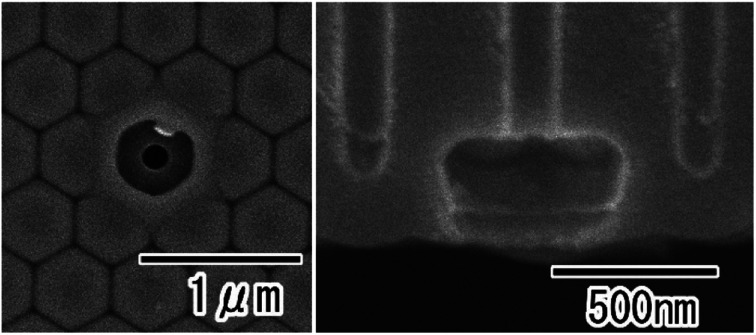

## Introduction

The preparation of through-hole membranes containing uniform-size holes with submicron to nanometer scales is important because they can be used in various applications, including filters, biosensors, and templates.^1–12^ Anodic porous alumina, which is formed by the anodization of Al in an acidic solution, is a typical material with an ordered through-hole array. This material has a unique geometrical structure composed of closely packed hexagonal cylinders with central holes. One of the characteristic features of anodic porous alumina is the good controllability of its geometrical structures *via* the anodization parameters. The hole interval and hole size are dependent on the voltage applied for anodization. Pore-widening treatment after the anodization can be applied to adjust the hole size. In addition, the capability of forming a long-range ordered hole arrangement under appropriate anodizing conditions is an advantageous feature of anodic porous alumina as a functional material.^13^ A through-hole membrane of anodic porous alumina is usually obtained by the selective dissolution of an Al substrate and subsequent removal of the bottom part of the oxide called the barrier layer.^14–16^

In some application fields, the site-controlled selective through-holing of anodic porous alumina is required. For example, the selective through-holing of anodic porous alumina followed by the deposition of materials into the through-holes can be performed to introduce defects in photonic crystals, which act as sites for the confinement of light. Previously, selective through-holing has been carried out by the selective etching of a barrier layer using a focused ion beam (FIB).^17–19^ The problem of through-holing by a FIB is its low throughput in the fabrication. As an alternative process, a process of selective through-holing based on the pretexturing of Al has been developed.^20,21^ In this process, the texturing of Al using a mold with an ordered array of convexes and defects at periodic sites enabled the preparation of anodic porous alumina with controlled through-hole sites. The selective through-holing was achieved by exploiting the differences in the thickness of the barrier layer at the imprinted and non-imprinted sites of the Al substrate. The barrier layer was thinner at the non-imprinted sites than at the imprinted sites because of the differences in the growth rates of the holes during anodization. The holes formed at non-imprinted sites could be through-holed selectively by wet etching. However, this process also had several problems. For example, the preparation of large membranes with selective through-holes was difficult because the molds used for the pretexturing of Al were formed by electron beam lithography, for which the formation of fine patterns with a large area was difficult. In addition, selective through-holing membranes with high aspect ratios could not be obtained owing to the difficulty in maintaining the ordered hole arrangement with defects during the anodization.

In the present report, we describe a new process for the preparation of anodic porous alumina with site-controlled through-holes. This process is based on the formation of a masking layer on anodic porous alumina, and subsequent selective second anodization to form a readily soluble layer. An advantage of this process is the controllability of the pattern of through-holes by changing the mask pattern. In addition, the preparation of large samples with selective through-holes is possible. This is because that the method reported here allows the formation of multiple selective through-holes simultaneously regardless of the number of through-holes by a single processing. The selective through-hole membrane with a size of 1 cm^2^ or more could be obtained by the present process. This is the first report on the formation of large alumina membranes with through-hole sites controlled by a wet process, which is different from the conventional process using dry etching. The obtained through-hole membranes can be used in various functional devices requiring controlled through-hole sites.

## Experimental

**Fig. 1:**
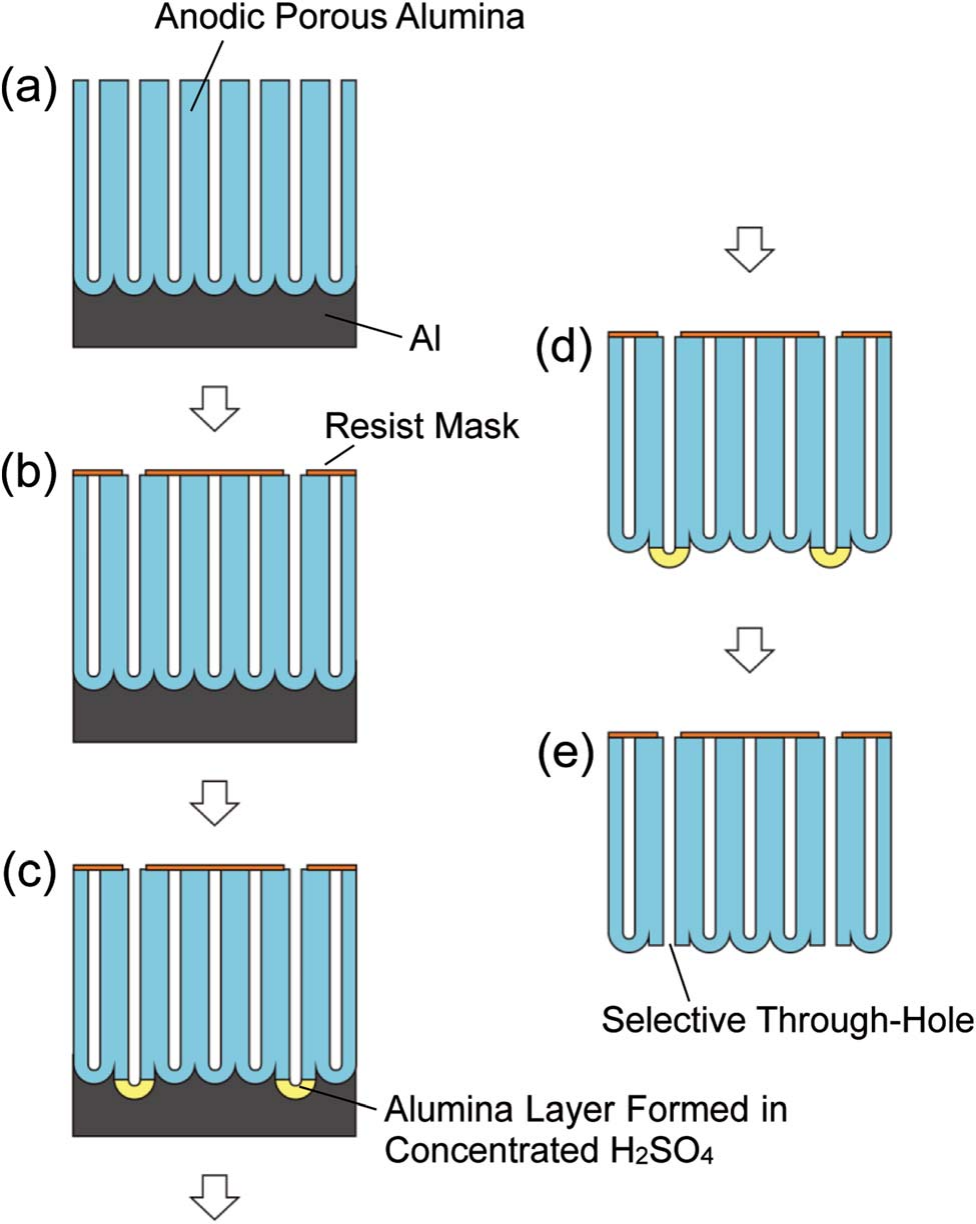
Schematic diagram of fabrication of anodic porous alumina membrane with controlled through-hole sites: (a) preparation of anodic porous alumina, (b) formation of a resist mask on the surface of the anodic porous alumina, (c) anodization in concentrated sulfuric acid, (d) removal of the Al substrate, and (e) selective through-holing by wet etching.

Fig. 1 shows a schematic diagram of the preparation of an alumina through-hole membrane with controlled through-hole sites. In the present study, ideally ordered anodic porous alumina prepared by the pretexturing of Al was used as a starting material.^22^ Before the anodization, an ordered concave array, which provided the initiation sites for hole development during the anodization, was formed on the surface of an Al sheet by pretexturing using a Ni mold with an ordered convex array with 500 nm intervals. The pretextured Al was anodized at a constant voltage of 200 V in 0.1 M phosphoric acid at 0 °C for 90 min. To form a resist mask on the surface of the ideally ordered anodic porous alumina, a polydimethylsiloxane (PDMS) stamp, which was formed using a master mold fabricated by electron beam lithography, was used. A thin polychloroprene layer was formed on the surface of the PDMS stamp by dip-coating using a toluene solution containing 0.75 wt% polychloroprene. The PDMS stamp with a thin resist layer was pressed on the porous alumina to transfer the resist pattern to the surface of the alumina layer. Then, the anodic porous alumina with the resist mask was anodized again under a constant voltage of 200 V in 17.6 M sulfuric acid at 0 °C. This selectively formed a highly soluble alumina layer at the bottoms of the holes in the open sites of the resist mask. After the anodization, the residual Al substrate was removed using a saturated solution of iodine in methanol, and the highly soluble alumina layer formed in concentrated sulfuric acid was dissolved selectively by wet etching using 10 wt% phosphoric acid at 30 °C for 5 min. The obtained samples were observed by scanning electron microscopy (SEM; JSM-6700F, JEOL).

## Results and discussion

**Fig. 2:**
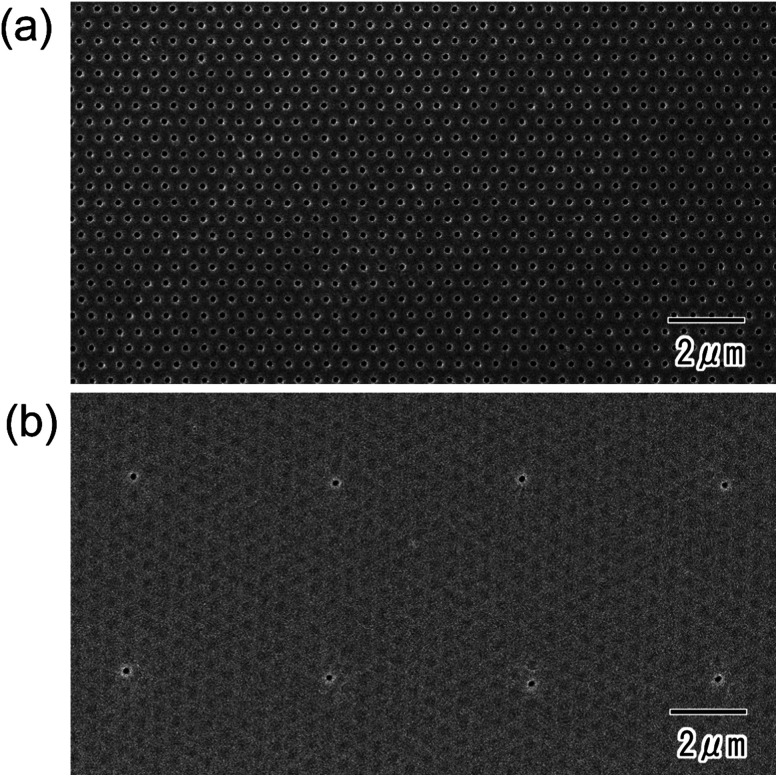
SEM images of anodic porous alumina before (a) and after (b) formation of the resist mask.

Fig. 2 shows surface SEM images of the ideally ordered anodic porous alumina before (Fig. 2(a)) and after (Fig. 2(b)) the formation of the resist mask using the PDMS stamp with a square array of convexes. The convexes in the PDMS stamp were spaced at 5 μm intervals and each had a diameter of 300 nm. A hexagonal array of uniform holes in anodic porous alumina was observed (Fig. 2(a)). For the determination of the average hole interval and diameter of the anodic porous alumina, the interpore distances and diameters of 100 holes were measured from SEM observation. The average vales were calculated form the measured values. The hole interval and diameter of the sample were 500 and 130 nm, respectively. The relative standard deviations of those values were less than 5%. The ideally anodic porous alumina shown in Fig. 2 was prepared by pretexturing process of Al using the mold with an ordered convex array with 500 nm intervals. The hole interval of obtained anodic porous alumina was in good agreement with the convex interval of the Ni mold. The surface SEM image (Fig. 2(b)) shows that single openings were arranged in a square pattern with 5 μm intervals, and the other holes in the anodic porous alumina were covered by the resist mask. This means that the thin resist mask was transferred from the surface of the PDMS stamp to the porous alumina by intaglio printing.

**Fig. 3:**
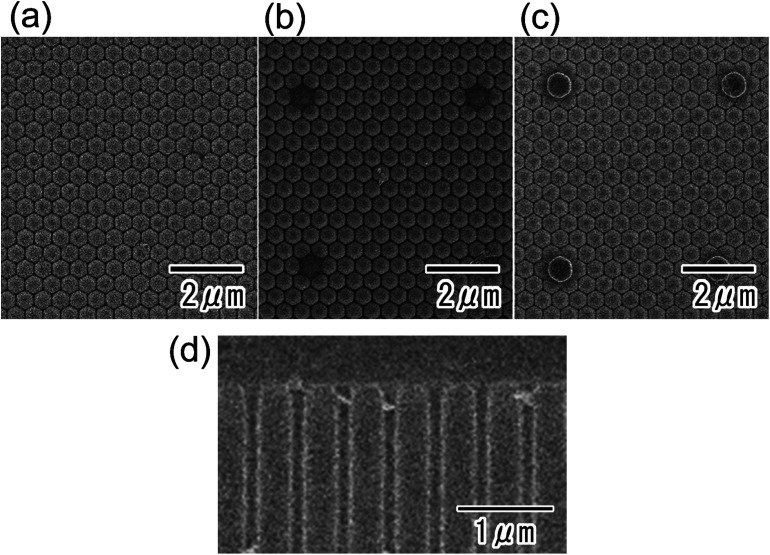
SEM images of the back surface of anodic porous alumina before (a) and after anodization in concentrated sulfuric acid for 270 min (b) or 360 min (c). (d) Cross-sectional SEM image of the surface of anodic porous alumina after the anodization in 17.6 M sulfuric acid for 360 min.

SEM images of the back surface of the anodic porous alumina were also obtained before (Fig. 3(a)) and after the anodization in 17.6 M sulfuric acid for 270 min (Fig. 3(b)) or 360 min (Fig. 3(c)). In the present study, a 17.6 M sulfuric acid was adopted to realize the stable anodization without the excess current, which destroy the oxide film due to the burning. The anodization in a very high concentrated sulfuric acid allowed a stable anodization of the samples even under high anodizing voltage conditions because the solution resistance of a very high concentrated sulfuric acid is low.^23^ The average current density of the samples during the anodization in 17.6 M sulfuric acid was less than 1 mA cm^−2^. For the SEM observation, the residual Al substrate was dissolved in a saturated iodine solution. Before the anodization in 17.6 M sulfuric acid, hexagonal alumina cells with uniform sizes and contrast were observed (Fig. 3(a)). However, after the anodization, alumina cells that projected from the surface were observed at 5 μm intervals. The interval and arrangement of these cells agreed with those of the openings of the resist mask formed on the top of the sample. The sizes of the projecting alumina cells increased as the anodization time increased because the anodization in concentrated sulfuric acid proceeded selectively at the opening sites of the resist mask formed on the top of the sample. Fig. 3(d) shows the cross-sectional SEM image of the surface part of anodic porous alumina after the anodization in 17.6 M sulfuric acid for 360 min. From the SEM image, it was confirmed that the ordered hole array structure was maintained even after the anodization in a very high concentrated sulfuric acid.

**Fig. 4:**
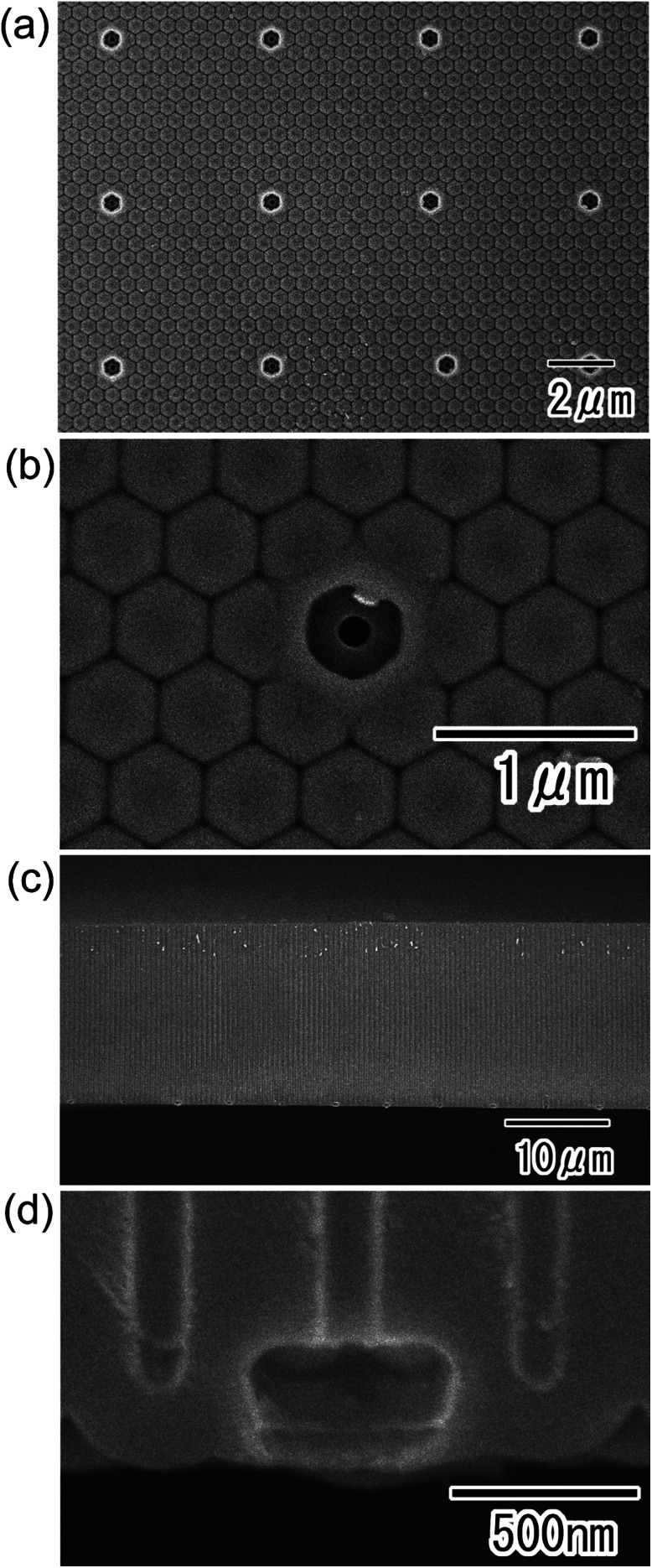
SEM images of the anodic porous alumina membrane with controlled through-hole sites. (a) Low-magnification and (b) high-magnification views of the back surface of the membrane. (c) Low-magnification and (d) high-magnification cross-sectional views of the membrane.

Next, SEM images of the porous alumina membrane were obtained after etching in 10 wt% phosphoric acid for 5 min (Fig. 4). Low- (Fig. 4(a)) and high-magnification (Fig. 4(b)) images of the back surface of the sample showed that selective through-holing was achieved by selective dissolution of the alumina layer formed in concentrated sulfuric acid. Cross-sectional SEM images (Fig. 4(c) and (d)) confirmed that straight through-holes with depths of 20 μm were arranged at uniform intervals of 5 μm. The through-hole diameter was 130 nm even after etching because the alumina layer formed in phosphoric acid was not dissolved during the etching treatment.^16^ In the present study, the resist mask was not removed and remained on the surface of the anodic porous alumina even after anodization in 17.6 M sulfuric acid. However, the resist mask could also be removed by heat treatment at 700 °C for 1 h after the selective through-holing. The thickness of the obtained porous alumina membrane shown in Fig. 4 was 20 μm. The obtained membrane had mechanical strength enough to handle with tweezers. The selective through-holing membrane with a thickness of more than 20 μm could also be obtained by adjusting the anodization time.

**Fig. 5:**
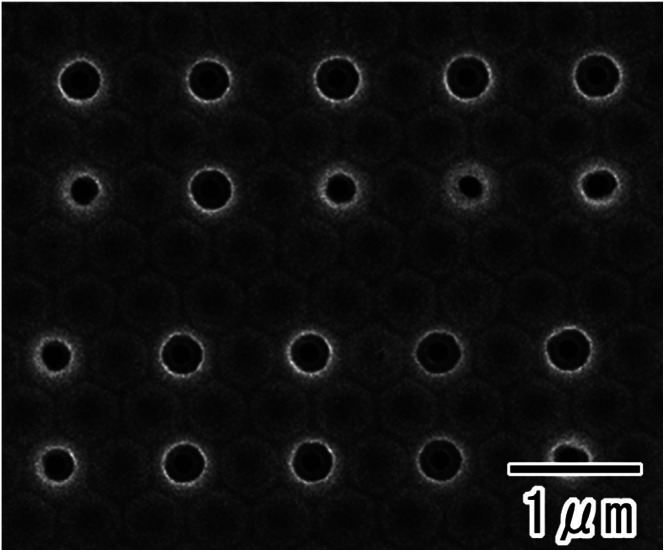
SEM image of the back surface of an alumina membrane with selective openings with 1 μm intervals. The anodization in 17.6 M sulfuric acid was performed for 360 min.

The interval between the selective openings could be controlled by changing the pattern of the resist mask formed on the surface of the anodic porous alumina. Fig. 5 shows the back-surface image of a sample prepared by this process using a PDMS stamp with a square pattern and 1 μm intervals. The SEM image (Fig. 5) confirmed that selective openings were formed at every other hole in anodic porous alumina with 500 nm intervals.

**Fig. 6:**
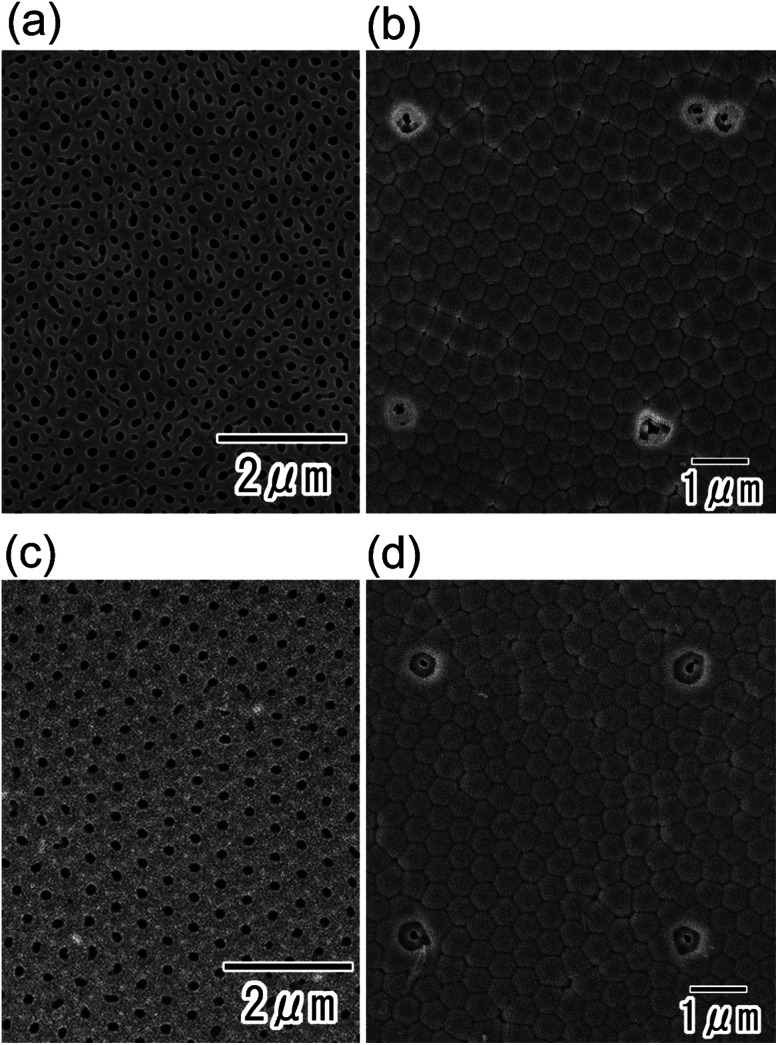
(a) Top surface image of disordered anodic porous alumina before formation of the resist mask. (b) Image of the back surface of disordered anodic porous alumina after selective through-holing by the present process. The sample was formed by the anodization of Al substrate in 0.1 M phosphoric acid at 0 °C under a constant voltage (195 V). For the selective through-holing, the anodization in 17.6 M sulfuric acid was performed for 360 min. (c) Image of the top of self-ordered anodic porous alumina prepared by a two-step anodization process. The first and second anodizations of Al were performed in 0.1 M phosphoric acid at 0 °C under a constant voltage (195 V). (d) Back surface image of self-ordered anodic porous alumina after selective through-holing by the present process. For the selective through-holing, the anodization in 17.6 M sulfuric acid was performed for 360 min.

Fig. 6 shows the results of applying this process to disordered anodic porous alumina (Fig. 6(a)) and self-ordered anodic porous alumina (Fig. 6(c)). These samples were 20 μm thick. In the disordered anodic porous alumina (Fig. 6(b)), the arrangement of the selective openings was disturbed and two or three openings formed each other. This is because the holes in the disordered anodic porous alumina became bent or branched during anodization. By contrast, in the self-ordered anodic porous alumina (Fig. 6(d)), the ordered square arrangement in the resist mask formed on the top of the sample was maintained at the bottom of the anodic porous alumina. This is because straight holes formed between the surface and the bottom of the self-ordered anodic porous alumina. Under an appropriate anodizing condition, an ordered nanohole array structure with straight cylindrical holes were formed self-organizingly.^24^ From these results, we concluded that an ordered array of selective openings could be obtained even in self-ordered anodic porous alumina with a multiple domain hole arrangement.

**Fig. 7:**
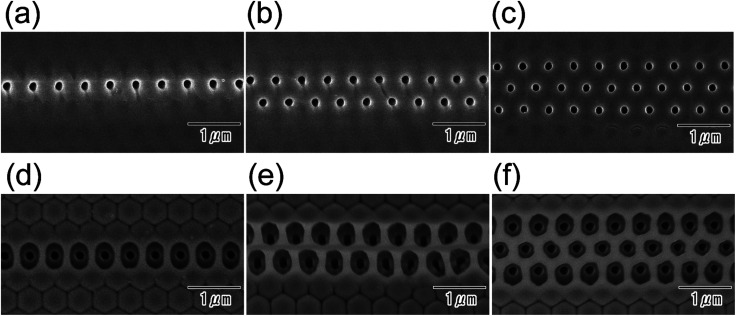
Images of the top of anodic porous alumina after the formation of resist mask having line patterns with different widths between openings: (a) 300 nm, (b) 600 nm, and (c) 900 nm. Images of the back surface of anodic porous alumina after selective through-holing with opening widths in the resist masks of (d) 300 nm, (e) 600 nm, and (f) 900 nm. For the selective through-holing, the anodization in 17.6 M sulfuric acid was performed for 360 min.

The pattern of selective openings could be controlled by changing the pattern of the resist mask. Fig. 7(a)–(c) show SEM images of surfaces of anodic porous alumina with a resist mask of line patterns. The width of the openings in the line pattern of the resist mask was controlled to 300 nm (a), 600 nm (b), or 900 nm (c) by changing the master pattern formed by electron beam lithography. Images of the back surfaces (Fig. 7(d)–(f)) showed that the selective openings were arranged in a straight line. The number of rows of selective openings was controlled *via* the width of the openings in the line pattern in the resist mask. From these results, we concluded that the pattern of selective openings in an alumina membrane can be precisely controlled using the developed process.

**Fig. 8:**
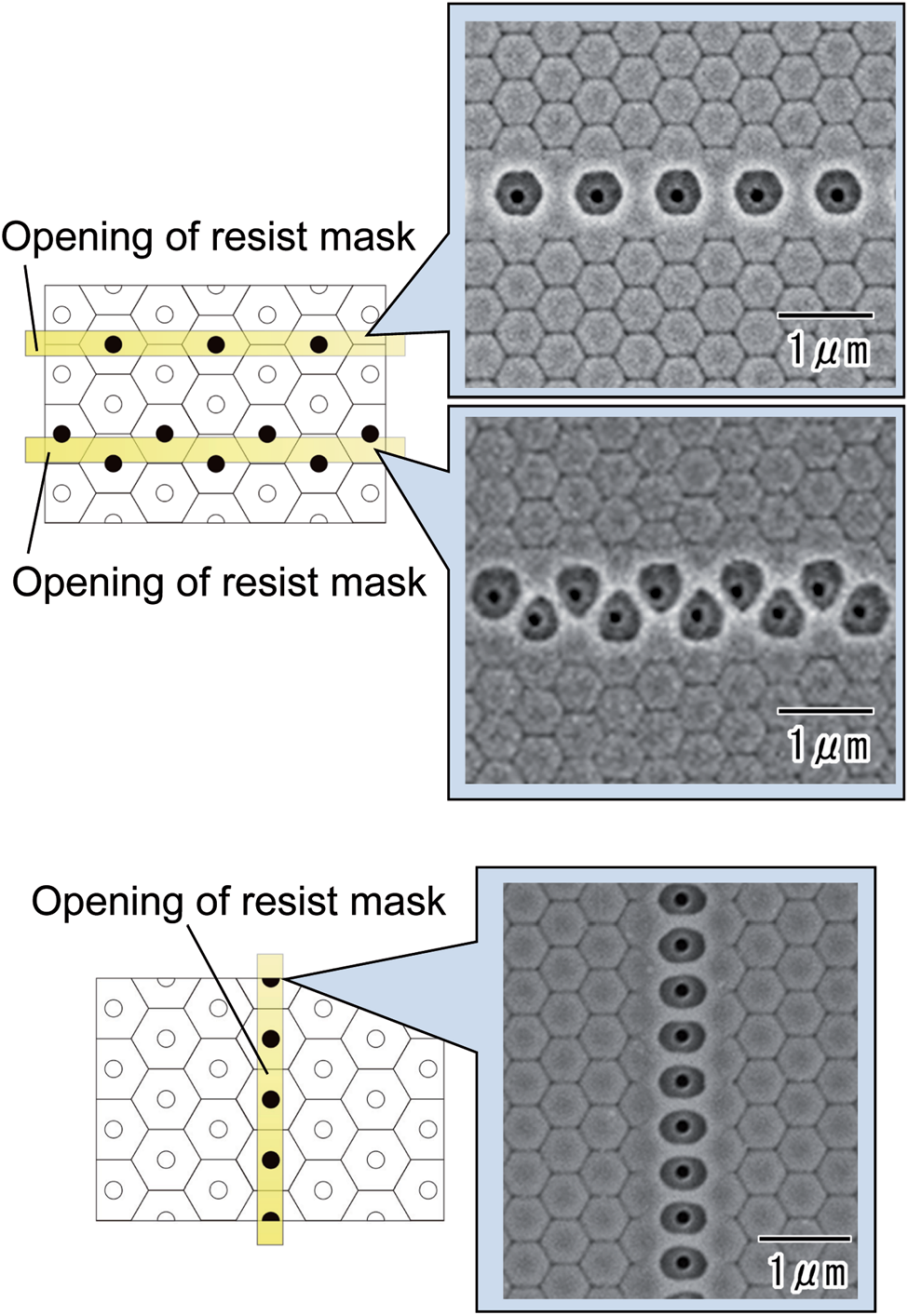
Control of the arrangement of selective through-holes by adjusting the alignment of linear openings in the resist mask and the orientation of the hole arrangement.

The linear arrangement of openings could also be controlled by adjusting the direction of the hole arrangement in the ordered anodic porous alumina. SEM images of the back surfaces (Fig. 8) of anodic porous alumina ware taken after selective through-holing. The interval between the holes in the ideally ordered anodic porous alumina was 500 nm. The width of the line pattern in the resist mask formed on the top of the anodic porous alumina was 400 nm. The SEM images (Fig. 8) confirmed that aligned and offset openings could be obtained by adjusting the alignment of the line pattern in the resist mask and the orientation of the hole arrangement.

## Conclusions

Anodic porous alumina membranes with controlled through-hole sites were formed. To form a highly soluble alumina layer at the bottom of selected holes, a resist mask was formed on the surface of anodic porous alumina, and the sample was anodized in concentrated sulfuric acid. After the removal of the Al substrate, the alumina layer formed in concentrated sulfuric acid could be dissolved selectively by wet-etching in a phosphoric acid solution. The arrangement and interval of the through-holes could be controlled by changing the pattern of the resist mask on the surface of the anodic porous alumina. In the case of using the line pattern mask, the number of rows of selective openings was controlled *via* the width of the openings in the line pattern in the resist mask. The present process could also be applied to self-ordered anodic porous alumina with a multiple domain hole arrangement. These porous alumina membranes with selective through-holing will be useful in various applications, such as filters, biosensors, and templates.

## Conflicts of interest

There are no conflicts to declare.

## Supplementary Material
